# Therapeutic Aquatic Exercise in Pregnancy: A Systematic Review and Meta-Analysis

**DOI:** 10.3390/jcm11030501

**Published:** 2022-01-19

**Authors:** José Maria Cancela-Carral, Benigna Blanco, Adriana López-Rodríguez

**Affiliations:** 1 Faculty of Education and Sport Sciences, University of Vigo, 36005 Pontevedra, Spain; beni_blanun@hotmail.com; 2 HealthyFit Research Group, Galicia Sur Health Research Institute (IIS Galicia Sur), Sergas-University of Vigo, 36213 Vigo, Spain; adrianalpez102@gmail.com

**Keywords:** body mass index, health, perception of effort, swimming, weight

## Abstract

This systematic review and meta-analysis aimed to assess evidence on the effects of aquatic exercise in pregnant women. The search included the following databases: Medline-PubMed, Web of science, PEDro, Scopus and SPORTDiscus databases. Seventeen randomized controlled trials were included (*n* = 2439, age 20–39 years; 31.30 ± 1.30 years). The systematic review carried out has indicated that aquatic exercise in pregnant women appears to have positive effects on preventing excessive maternal weight gain, improving maternal body image, as well as promoting healthy behavior, decreasing medical leave due to lower back pain during pregnancy, preventing gestational depression by improving maternal glucose tolerance levels, and reducing O’Sullivan test values. The Physiotherapy Evidence Database was used to evaluate the quality of the methodology of the selected studies, which were found to present an average methodological quality (PEDro scale: 5.05 points). Meta-analysis showed that aquatic exercise in pregnant women appears to have positive effects in the prevention of excessive maternal weight gain (mean difference −1.66 kg, 95% CI −2.67 to −0.66) and also to reduce birth weight mean differences (−89.13 g, 95% CI −143.18 to −35.08). The practice of aquatic exercise is appropriate throughout pregnancy. However, more research is needed to build more solid knowledge on the benefits of aquatic physical exercise on physical fitness (endurance, flexibility, agility and strength).

## 1. Introduction

Pregnancy is a key life process, experienced by many women, which causes anatomical, physiological, metabolic, morphological and psychological modifications. All of these changes are continuous and gradual, allowing the pregnant woman to adapt to them progressively, and thus facilitate the proper development of the fetus, and her own preparation for childbirth, postpartum and lactation [1]. However, at varying percentages, the following can be associated with pregnancy: hormonally caused pathologies: gingivitis (35–50%) [2], constipation (11–40%) [3], hyperemesis gravidarum (0.3–2%) [4]; behavioral pathologies: gastroesophageal reflux (30–50%) [5] or due to the physiological changes in pregnancy: anemia (14%) [6]. Heart Disease induced by pregnancy is very rare, while the development of any type of cardiovascular disease in pregnancy occurs in between 1–4% of all pregnancies [7,8].

The incorporation of women into the workplace dates back to the time of the Second World War, due the need for them to carry out the work that had previously been performed by men, who were were now at the front. Increasing the number of women in the workplace has led many of them working during their pregnancies and breastfeeding periods, which in turn increases the risks that can affect working mothers throughout their productive lives [9].

The regular and systematized practice of physical exercise means that the risks, pathologies and modifications that pregnant women experience have less impact on their health and daily work, and on the health of their fetuses [10,11,12]. In recent years, new trends in physical exercise have emerged for pregnant women such as Pilates [13], yoga [14], tai Chi [15], low or moderate intensity aerobic exercise [16], but the ones that stand out among these are programs of physical exercise carried out in the aquatic environment [17,18,19,20,21,22,23,24,25,26,27,28,29,30,31,32,33].

Aquatic physical exercise aimed at pregnant women has proliferated in recent years, thanks to the benefits provided by the aquatic environment, such as a decrease in gravitational pull, an improved sense of physical comfort, improved mobility and flexibility, reduction of post-exercise pain [34] and an improvement of venous return due to the increased hydrostatic pressure [35]. However, these aquatic programs are characterized by their heterogeneity and by containing very diverse content (calisthenics, strength, aerobics, flexibility), and the effects that these can have on pregnant women are unknown. Therefore, the aim of this systematic review and meta-analysis is to identify the effects generated by different aquatic physical activity programs available for pregnant women on their physical fitness, anthropometric and cognitive components.

## 2. Materials and Methods

### 2.1. Search Strategy and Screening Procedures

A systematic search of published studies was conducted up to February 2020. Searches were carried out on the following electronic databases: MEDLINE-PubMed (1980–present), Scopus (1980–present), Web of Science (1982–present), PEDro (1980–present) and SPORTDiscus (1980–present). A specific search of the Cochrane Library was carried out to exclude the existence of revisions which had the same goal as the present one. The following search criteria were applied: “pregnancy” OR “pregnant” AND “aquatic exercise” OR “aquatic/water therapy” OR “swimming” OR “aquatic physical activity” AND “intervention*” OR “trial” OR “randomized controlled trial”.

### 2.2. Inclusion/Exclusion Criteria

The aim of this review was to analyze published studies that investigate the effects of aquatic therapy programs on the fitness, anthropometric and cognitive components of pregnant women.

On the basis of the titles of the articles and summaries, reports were prepared identifying inclusion/exclusion criteria. The inclusion criteria that the papers had to meet were (1) topics: pregnancy, (2) participants: pregnant women (age: 31.30 ± 1.30 years; gestational age: third trimester (70.58%); women experiencing their first pregnancies: 88.23%; comorbidities: 41.16%), (3) physical activity program: aquatic/water, (4) study design: randomized controlled clinical trial (RCT), (5) variables to study: fitness, anthropometric and cognitive.

### 2.3. Selection Process

Two reviewers (B.B. and J.C.) independently read all the summaries and classified them as excluded or potentially included. A third reviewer (A.L.) was asked for their opinion if there was a need to resolve any disagreement between the two reviewers. The studies were selected based on their titles and summaries. When the summaries were relevant for the purpose of the review, the entire article was read. The reviewers agreed to include 17 articles after reading them and rigorously applying the inclusion criteria.

### 2.4. Meta-Analysis

Statistical software Review Manager (RevMan) version 5.3 (Cochrane, London, UK) was used to analyze data. The meta-analysis focused on the maternal weight gain (*n* = 5), body mass index (*n* = 6) and birth weight (*n* = 9), because they were the most studied variables in the selected studies. The mean difference and standard error between intervention and control groups, as well as the sample size for both groups, were entered into Review Manager. A random-effects model was utilized for the meta-analysis. Heterogeneity was determined by examining the I^2^ and Q statistics, both provided as an output by Review Manager. All *p*-values were two-tailed with a level of significance of <0.05.

### 2.5. Quality Assessment

The selected studies were subjected to an analysis and evaluation of their methodological quality by the interviewers independently (B.B. and J.C). The methodological quality of the tests was assessed using the PEDro scale [36]. The PEDro scale aims to evaluate four fundamental methodological aspects of a study such as randomized processing, blinding technique, group comparison, and data analysis process. The PEDro scale is based on the Delphi list developed by Verhagen et al. [37], which includes 11 items: specified eligibility criteria (this item is not used to calculate the PEDro score), random assignment, hidden location, reference comparability, blinded participants, blinded therapists, blinded evaluators, proper follow-up, intent-to-treat analysis, group-to-group comparisons and point estimates and variability. The reliability of this scale was assessed with acceptable results in intra-class correlation coefficients (ICF) equal to 0.56 (95% CI 0.47–0.65) for the ratings of individuals and BCIs for consensus ratings equal to 0.68 (95% CI 0.60–0.76). The test quality assessment in the PEDro database was conducted by two trained independent evaluators (B.B and J.C.) and any disagreements were resolved by a third evaluator (A.L.). PEDro scale scores ranged from 1 to 10, with higher PEDro scores corresponding to higher study quality. The following criteria were used to rate the quality of the method: A PEDro score of less than 5 indicates low quality and a PEDro score of 5 or higher indicates high quality [31].

## 3. Results

**Figure 1:**
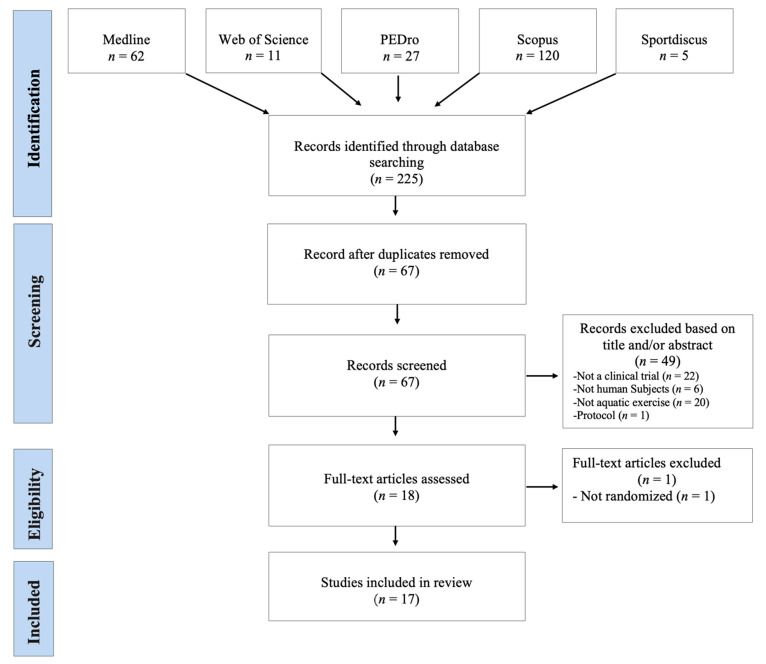
Flowchart of the included studies (PRISMA).

The database search identified 225 records. After the elimination of duplicates, 67 records were selected for their relevant content. During the analysis of the title and summary, 49 articles were excluded. Eighteen potentially relevant full-text articles were evaluated and one of them was excluded due to the lack of a control group (not RCT). Therefore, a total of 17 studies were included in this revision (Figure 1). Data were collected ethically and in ways which addressed the research questions. All the studies produced valuable research with clear statements of findings.

### 3.1. Description of Studies Include in Review

From the selected studies, a total of 2439 pregnant women were studied (Table 1). Regarding the size of the samples used, the study of Barakat et al. stands out [21] for being made up of 568 pregnant women, while in the rest of the studies the samples were smaller, made up of fewer than 270 pregnant women. All the studies used different samples except for Rodríguez-Blanque and collaborators who contributed to the review with four articles in which different variables were studied with the same sample [25,26,27,28]. The average age of the pregnant women who participated in the studies was 31.30 ± 1.30 years, with the Smith and Michel [31] study standing out for having the lowest average age of 25.10 ± 4.40 years. As for the number of weeks of gestation, all studies began the intervention in the 20th or subsequent week. The study conducted by Sillero et al. [30] is the only one that had a later start (31st week).

The types of intervention undertaken by the experimental group were characterized by aerobic, resistance or callisthenic exercises, which were carried out in the aquatic environment. However, in four studies the experimental group undertook a program that combined water intervention with land intervention [20,21,22,23]. With regard to the activity performed by the control group, it should be indicated that in 13 studies routine prenatal care was undertaken, while the remaining four carried out land-based physical activity [18,24,30,31]. The aquatic programs had a minimum duration of 6 weeks [33] and a maximum of 34 weeks [20]. As for the duration of the sessions it should be noted that they were all in the 45–60 min time zone, with the most common weekly frequency being of three sessions. Only one study had a different intervention length, of 25 min [18]. The pregnant women’s adherence to aquatic programs was on average 94.52%, this being higher than that of the land based program, where there was an adherence of 87.30%.

The intensity of the aquatic exercise undertaken was measured using the Borg scale, with the most common effort value being Level 12; [18,19,20,22,23,25,26,27,28,29]. In other studies, the effort made by the pregnant women was quantified and measured via their heart rate [18,20,22,23,25,26,29,33]. Other parameters analyzed in the 17 selected studies were: BMI, through the Quetelet formula [17,19,23,25,26,28], the Global Physical Activity Questionnaire test (GPAQ) to measure the level of physical activity [27,29], the cardiac response to effort [18], the effect of physical exercise on blood glucose level [13,15,16], maternal and baby weight measurements [19] and gestational and postpartum weight [29].

The results obtained from the different studies analyzed show that the programs carried out in the aquatic environment generated greater improvements in the majority of the variables suited to objective comparison than those conducted in the land environment or in the combined environment (water–land) (Table 1).

### 3.2. Quality Assessment

The range of values on the PEDro scale was one to ten. Five studies obtained less than five with the rest of the studies (*n* = 12) obtaining an average of 5.05 points on the PEDro scale of 5.05, which indicates that the methodological quality of the studies analyzed is not high. The year of publication does not seem to be an element that influences the quality of studies, as there are low quality studies published in 2006 and 2012, and average quality studies published between 2006 and 2019 (see Table 2). The most common criteria are issues related to the statistical procedure such as: “point measure and variability”, “between groups comparisons” and “random allocation” (*n* = 16) and also with “groups Similar at baseline” (*n* = 14). The criteria “blinded participant”, “blinded therapist” and “intention-to-treat analysis” were only found in one of the studies analyzed, while the criterion “blinded assessor” appeared in two (Table 2).

### 3.3. Meta-Analysis

The meta-analytical process was carried out on the most studied variables from the selected documents, and for which data were available. The variables studied in the meta-analysis were maternal weight gain (Kg), body mass index (m/kg^2^) and birth weight (g).

**Figure 2:**
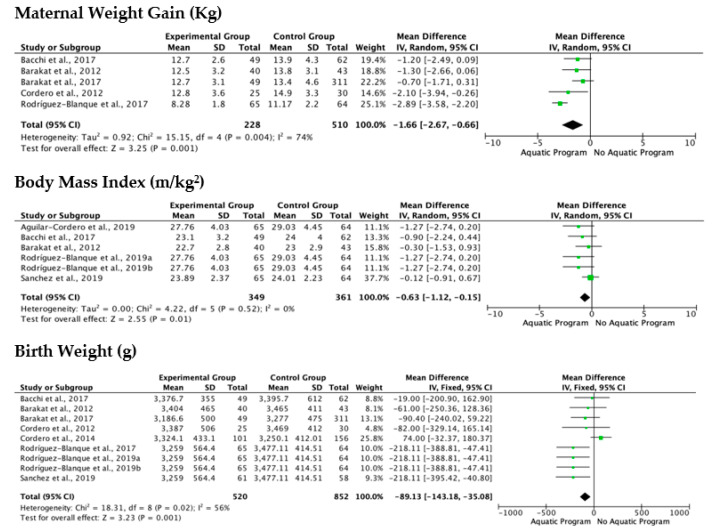
Forest plot of the mean overall (95%) of maternal weight gain (Kg), body mass index (m/kg^2^) and birth weight (g) for each study included in the meta-analysis. Obs: Green square in-dicates the mean difference in each study. Black diamond indicates the mean difference of all studies.

The analysis of the included studies showed a low level of heterogeneity based on I^2^ and Chi^2^ in the variable body mass index (m/kg^2^), while in maternal weight gain (Kg) and birth weight (g) had a high heterogeneity, being Chi^2^ = 15.15 and 18.31 respectively (Figure 2). The analysis of the effects of aquatic or combined water/land programs on maternal weight gain showed different trend, with particularly striking results coming from the Rodriguez-Blanque et al. study [25], in which a water-based program was compared to routine prenatal care, and where significant, improvements in the control of maternal weight were demonstrated by the water-based program.

The results of birth weight analysis show that both aquatic and non-aquatic exercise programs help regulate newborn weight, with no significant differences apparent between them (Figure 2). The study of Cordero et al. [23], was the only one that showed a trend leaning in favor of non-aquatic programs.

The last variable studied through meta-analysis was the body mass index (m/kg^2^), which was characterized by a low level of heterogeneity, demonstrating similar behavior in the Florest plot analysis throughout all of the studies analyzed, which reflects that the aquatic or combined programs have a positive effect on the reduction of the body mass index (Figure 2).

## 4. Discussion

This systematic review and meta-analysis aims to identify the effects of therapeutic aquatic exercise programs on the health (physical and cognitive) of pregnant women and newborns. The present study revealed that aquatic exercise programs help to control heart rate and blood glucose level, prevent excessive weight gain, while also improving balance and mobility in pregnant women.

The aquatic programs also help to control newborn weight, but the differences when compared to the non-aquatic programs are not significant [31].

Furthermore, on the cognitive variable, a positive effect was observed for postpartum depression, body image, as well as for the quality of life of pregnant women. Pregnancy is known to be an emotionally difficult period in which women can experience emotional ups and downs, these potentially manifesting from the first trimester [21]. The practice of physical exercise, and specifically exercise carried out in the aquatic environment helps in the control of these emotional and physical changes.

The type of physical intervention (aerobic, muscular resistance, calisthenics or routine prenatal care) together with the environment in which these exercises are carried out (aquatic or land), determine the results. It should be noted that programs containing a mixed intervention (aerobic + muscular resistance) and that combine the aquatic and land environment have a more positive effect on glucose levels [20,22], on the prevention and reduction of gestational diabetes [21,22,23] and on the reduction of maternal weight [20] than routine prenatal care programs. This is due to the greater energy demand involved in performing aerobic and muscular resistance training in water, where the resistance to movement is much higher than that which is experienced in routine prenatal care [20].

The intensity of each intervention program was registered by using Borg’s scale, or by means of a heart rate monitor. Regardless of which measurement tool was used, the studies included in this paper reflected a homogeneous work intensity (Borg’s scale; 12–14; heart rate monitor: 60–70% maximum HR), and did not generate differential effects in the cognitive and physical variables analyzed [17,20].

The duration of the intervention programs is a key parameter in producing the desired effects during pregnancy, with the minimum duration after which positive effects were demonstrated—in the cognitive (body image) and physical (balance, mobility) variables—being six weeks [31]. Each training session lasted between 45–60 min, with the average frequency being three times a week.

The analysis of the different aquatic physical exercise programs revealed that the programs with a greater volume of load produced greater benefits than those programs which had a greater intensity of load. This may be due to the increase in caloric expenditure and blood flow generated by activities of long duration but of low intensity, thus favoring the control of depression [17], body weight [18], gestational diabetes [21], glucose levels [22], lower back pain [24], sleep quality [26] and quality of life [32].

From the present study, carried out on the meta-analysis of the influence of aquatic vs. land-based physical exercise programs on maternal weight, BMI and newborn weight, it can be concluded that the aquatic program has a significant, positive effect on maternal weight control [19,20,21,22,25]. Regarding BMI and the newborn weight, the results indicate a positive trend leaning towards aquatic programs, but the differences demonstrated are not significant [19,20,23,29].

### Strengths and Limitations

The strengths of this study lie in the size of the sample (*n* = 2439 pregnant women) which was used, and the stability of intensity in the aquatic physical exercises carried out, which has allowed us to compare the different programs which were used. The limitations we encountered in this study are mainly due to the fact that there are still few studies that analyze the effect of aquatic physical exercise as compared to to land-based physical exercise, or other activities. Another limitation that needs to be addressed is the diversity of variables and assessment tools that the different studies presented, which made it difficult to compare the results. Therefore, it is necessary to carry out more randomized controlled experimental studies that reinforce the trends observed during the course of this systematic and meta-analysis review.

## 5. Conclusions

Undertaking this systematic review and meta-analysis has allowed us to conclude that the regular and systematic practice of aquatic exercise or combined land exercise carried out during pregnancy provides better control of the weight of both mother and newborn, as well as leading to improvements at the cognitive (depression, quality of life, body image) and physical level (low back pain, fitness, mobility) of the mother.

## Figures and Tables

**Table 1:** Key characteristics of studies (*n* = 17) included in the review.

Reference	Purpose	Participants	Mean Age ± SD (Median/Range)	Gestational Age (Weeks)	Type of Intervention (E/C)	Intervention (Wk/f/min)	Adherence	Findings	Measurement Tools
Aguilar-Cordero et al. [17]	Determine if physical activity in pregnancy relieves PPP.	EG = 65 CG = 64	EG = 34.52 ± 4.50 CG = 33.67 ± 5.37	20th–37th	EG = Aerobic and resistance activities-SWEP (water) CG = Routine prenatal care	EG = 17/3/60 CG = NR	EG = 92.82% CG = 91.4%	Less at risk of Depression (EPDS) in EG. Overweight and obesity are closely associated with PPD.	Perception of effort (Borg scale) BMI (Formula QUETELET) Depression postpartum (Edinburgh Postnatal Depression Scale-EPDS-)
Bacchi et al. [18]	Evaluate and compare maternal HR in water and land exercises with the same intensity	EG = 15 CG = 15	NR	3rd TRIMESTRE (27th–38th–42nd)	EG = Calisthenics exercise (water) CG = Calisthenics exercise (land)	EG = 1/1/25 CG = 1/1/25	EG = 100% CG = 100%	Calisthenics exercise (land) produce higher FC elevations (110.86 ± 6.10) than calisthenics exercise (water) (105.40 ± 6.10), but not significant differences.	Perception of effort (Borg scale) Heart rate monitor (Polar F6);
Bacchi et al. [19]	Study the effect of a program of aquatic activities on pregnancy on maternal weight and birth weight	EG = 49 CG = 62	EG = 30.4 ± 4.0 CG = 31.0 ± 5.0	10 to 12th/38 to 39th	EG = Aerobic and resistance activities (water) CG = Routine prenatal care	EG = 26–29/3/55–60 CG = NR	EG = 70% CG = 88.57%	Higher percentage of women with excessive maternal weight gain in the CG (45.2%; *n* = 28) than in the EG (24.5%). Aerobic and resistance activities (Water) increase maternal weight and preserves birth weight.	Perception of effort (Borg scale) BMI (Formula QUETELET)
Barakat et al. [20]	Analyze glucose tolerance through aquatic exercises for pregnant women	EG = 40 CG = 43	EG = 32 ± 4 CG = 31 ± 3	24th–28th	EG = Aerobic activities (land + water) CG = Routine prenatal care	EG = 32–33/2 + 1/35–45 CG = NR	EG = 80% CG = 86%	The glucose values corresponding to the EG (103.80 ± 20.40 mg/dL) were better (significant differences; *p* = 0.001) than those of the CG (126.9 ± 29.5 mg/dL). No differences in maternal weight and cases of gestational diabetes. Exercise during pregnancy improves the level of tolerance to maternal glucose.	Heart rate monitor (Accurex Plus, Sark Products, Waltham, MA, USA); Polar Electro OY (Polar, Kempele, Finland) Perception of effort (Borg scale) Blood glucose level (blood test)
Barakat et al. [21]	Compare terrestrial or aquatic exercises during pregnancy in maternal and neonatal outcomes.	EG1 = 107 EG2 = 49 EG3 = 101 CG = 311	EG1 = 31.5 ± 3.8 EG2 = 30.9 ± 4.0 EG3 = 32.0 ± 3.5 EC = 31.9 ± 4.5	9th–11th	EG1 = aerobic activities (land) EG2 = aerobic + resistance activities (water) EG3 = aerobic activities (land) + resistance activities (water) CG = Routine prenatal care	EG1 = 30/3/55–60 EG2 = 30/3/55–60 EG3 = 30/2 + 1/55–60 CG = NR	EG1 = 79.68% EG2 = 79.28% EG3 = 75.14% CG = NR	Exercise on land is more effective in preventing excessive maternal weight gain (*p* = 0.001). Combined programs or aquatic programs seem more effective in preventing gestational diabetes (*p* = 0.03). Both are safe for the baby.	Evaluate knowledge, attitudes and reasoning (questionnaire ad hoc) Height (stature meter) Weight (weight scale)
Cordero et al. [22]	Evaluate the effectiveness of a moderate exercise program during pregnancy on maternal weight, glucose and gestational diabetes.	EG = 25 CG = 30	EG = 34.1 ± 4.7 CG = 31.6 ± 2.0	6th–10th/38th–39th	EG = aerobic activities (land) + resistance activities (water) CG: Routine prenatal care	EG = 28/2 + 1/50 CG = NR	EG = 62.50%; CG = 75.00%	The exercise program performed during pregnancy reduced maternal weight gain (*p* = 0.03), values of the maternal glucose screen test (*p* = 0.002) and appears to prevent gestational diabetes.	Perception of effort (Borg scale) Heart rate monitor (Accurex Plus, Polar Electro OY). Blood glucose level (blood test)
Cordero et al. [23]	Assess the effectiveness of a maternal exercise program (land/aquatic activities) in preventing gestational diabetes mellitus.	EG = 100 CG = 146	EG = 33.6 ± 4.1 CG = 32.9 ± 4.5	10th–12th	EG = aerobic activities (land) + resistance activities (water) CG = Routine prenatal care	EG = 26–30/2 + 1/50–60 CG = NR	EG = 81.96% GC = 66.36%	The prevalence of GDM was reduced in the EG group (EG, 1%, *n* = 1, vs. CG, 8.8%, *n* = 13 (*p* = 0.009)). Exercise on land and in water reduced the incidence of DMG is associated with a decreased gestational weight gain and conserved glucose tolerance	Perception of effort (Borg scale) Heart rate monitor (Accurex Plus, Polar Electro OY) Blood glucose level (blood test) BMI (Formula QUETELET)
Granath et al. [24]	Evaluate and compare low back or pelvic and pain due to illness in pregnant women through terrestrial and aquatic exercises	EG = 132 CG = 134	EG = 29.10 ± 4.50 CG = 29.10 ± 4.50	11th–12th	EG = Aerobic activities (water) CG = Aerobic activities (land)	EG = 28–29/1/60 CG = 28–29/1/60	EG = 68.75% CG = 67.68%	Aerobic activities (Water) decreased low back pain related to pregnancy (*p* = 0.04) and sick leave (*p* = 0.03) more than a physical exercise program on land. Water exercises are recommended for pregnant women.	Pregnancy-related pelvic girdle pain (PLBP) Pregnancy-related low back pain (PPP)
Rodríguez-Blanque et al. [25]	Analyze the influence of a physical activity program in the aquatic environment on the newborn weight	EG = 65 CG = 64	EG = 34.52 ± 4.50 CG = 33.67 ± 5.37	20th–37th	EG = Aerobic and resistance activities-SWEP—(water) CG = Routine prenatal care	EG = 17/3/60 CG = NR	EG = 91.42%; CG = 92.85%	The aerobic and resistance activities-SWEP—(Water) doesn’t present birth risks premature and gestation time is not altered. The SWEP has achieved a significant decrease in the weight of the newborn and a lower weight gain during pregnancy.	BMI (Formula QUETELET) Perception of effort (Borg scale) Heart Rate Monitor (Quirumed OXYM2000, London, UK)
Rodríguez-Blanque et al. [26]	Determine if there is an association between physical activity in the aquatic environment and sleep quality in pregnant women.	EG = 65 CG = 64	EG = 32.12 ± 4.43 CG = 30.58 ± 4.75	20th–37th	EG = Aerobic and resistance activities-SWEP—(water) CG = Routine prenatal care	EG = 17/3/60 CG = NR	EG = 91.42%; CG = 92.85%	The SWEP methodology improves sleep quality, both subjectively and in terms of latency, duration and efficiency.	Evaluate BMI (Formula QUETELET) Perception of Effort (Borg scale) Heart Rate Monitor (Quirumed OXYM2000, London, UK) Evaluate self-perception (Pittsburgh Sleep Quality Index PSQI)
Rodríguez-Blanque et al. [27]	Determine the effect of a water exercise program on the rate of perineum intact after delivery.	EG = 65 CG = 64	EG = 32.12 ± 4.43 CG = 30.58 ± 4.75	20th–37th	EG = Aerobic and resistance activities-SWEP—(water) CG = Routine prenatal care	EG = 17/3/60 CG = NR	EG = 92.82%; CG = 91.40%	The women who followed the SWEP methodology were significantly more likely to have intact perinea after childbirth.	Physical activity level (Global Physical Activity Questionnaire, GPAQ) Perception of effort (Borg scale)
Rodríguez-Blanque et al. [28]	Determine the duration of labor in pregnant women who completed a program of moderate physical exercise in water and subsequently presented eutocic birth.	EG = 65 CG = 64	EG = 32.12 ± 4.43 CG = 30.58 ± 4.75	20th–37th	EG = Aerobic and resistance activities-SWEP—(Water) CG = Routine Prenatal Care	EG = 17/3/60 CG = NR	EG = 92.82%; CG = 91.40%	Women who exercised in water during pregnancy have a shorter duration of labor than those who did not. The difference was especially marked with respect to the duration of the first and second stages of labor (*p* < 0.001).	Perception of effort (Borg scale) Heart rate monitor (Quirumed OXYM2000) Total duration (minutes) of labor (Ad hoc questionnaire)
Sánchez García et al. [29]	Analyze the evolution of weight, gestational and postpartum, in pregnant women who perform an aquatic program.	EG = 65 CG = 64	EG = 32.12 ± 4.43 CG = 30.58 ± 4.75	20th–37th	EG = Aerobic and resistance activities-SWEP—(water) CG = Routine prenatal care	EG = 17/3/60 CG: NR	EG = 93.84%; CG = 90.60%	The SWEP methodology during pregnancy helps to control gestational weight gain and weight recovery before pregnancy.	Body weight (calibrated scale) Height (calibrated metal rod) BMI (Formula QUETELET) Physical activity level (Global Physical Activity Questionnaire, GPAQ) Perception of effort (Borg scale)
Sillero et al. [30]	Analyze the effect of two physical activities on skin temperature in women 31 weeks pregnant.	EG = 14 CG = 14	EG = NR CG = NR	31st	EG = Swimming; CG = Yoga	EG = NR CG = NR	EG = 100%; CG = 100%	Significant reduction in skin temperature of pregnant mothers after aquatic activity, in the areas of the mother and belly, in case of an inadequate water temperature. Tsk values are not dangerous for the fetus.	Thermograms (T335FLIR infrared camera)
Smith and Michel. [31]	Evaluate the impact of a water exercise program on the perception of body image, participation in health behaviors, participation in health promotion, level of physical discomfort and mobility.	EG = 20 CG = 20	EG = 25.10 ± 4.90 CG = 24.80 ± 5.60	19th	EG = Calisthenics exercise (water); CG = Normal activity of daily living	EG = 6/3/60 CG = NR	EG = 100%; CG = 100%	Water exercise can improve physical functioning, decrease maternal discomfort, improvement of the maternal body and improve health behaviors.	Body image (Prenancy Body shape Questionnaire, PBSQ) Effects of aquatic exercise (Pender’s Health Promotion Lifestyle Profile (HPLP)). Mobility (timed get up and go test) Physical discomfort (Smith’s Prenancy Discomfort Intensity Index (SPDII))
Vallim et al. [32]	Analyze the quality of life (QOL) in sedentary pregnant women through aerobic physical exercises in water	EG = 31 CG = 35	EG = 26 CG = 24	28th–36th	EG = Aerobic activities (Water) CG = Routine prenatal care	EG = 28–36/3/50 CG: NR	EG = 64.52% CG = 65.72%	The majority had eight or more years of schooling: 52% (EG) and 83% (CG), this difference being statistically significant (*p* = 0.0065).	Quality of life (Questionnaire WHOQOL-BREF)
Vázquez-Lara et al. [33]	Examine the effect of physical activity in the aquatic environment on hemodynamic constants in pregnant women.	EG = 18 EC = 28	EG = 31.0 ± 4.6 CG = 29.5 ± 6.1	25th–27th	EG = Calisthenics exercise AEPPW (water) CG = Routine prenatal care	EG: 6/2/45 CG: NR	EG = 90% CG = 100%	An aquatic exercise programme for pregnant women (AEPPW), contributes to the hydrosaline balance, preventing the excessive increase in the usual plasma volume (*p* < 0.010), increasing the secretion of sodium (0.050) and reducing the arterial pressures (*p* < 0.050)	Blood pressure monitor (Riester, Jungingen, Germany), Calculate plasma volume (Dill y Costill’s) Blood and urine test Heart rate monitor (Polar F4, Kempele Finland))

NR: not reported, SWEP: study water exercise pregnant; BMI: body mass index; PPD: postpartum depression; EG: experimental group; CG: control group; AEPPW: Aquatic Exercise Programme for Pregnant Women; HR: heart rate; GDM: gestational diabetes mellitus; PLBP: pregnancy related pelvic girdle pain; PPP: pregnancy related low back pain; PSQI: Pittsburgh Sleep Quality Index; GPAQ: global physical activity questionnaire; HPLP: Pender’s Health Promotion Lifestyle Profile; WHPQOL-BREF: The World Health Organization Quality of Life; QOL: quality of life; SPDII: Smith’s Pregnancy Discomfort Intensity Index.

**Table 2:** PEDro Methodology quality for studies (*n* = 17) included in the review.

Reference	Eligibility Criteria *	Random Allocation	Concealed Allocation	Groups Similar at Baseline	Blind Participant	Blind Therapist	Blind Assessor	Follow-Up	Intention to Treat Analysis	Between-Group Comparisons	Point Measure and Variability	PEDro Score Total
Aguilar-Cordero et al. [17]	1	1	0	1	0	0	0	1	0	1	1	5
Bacchi et al. [18]	0	1	0	1	0	0	0	1	0	1	1	5
Bacchi et al. [19]	1	1	1	1	1	1	1	1	1	1	1	10
Barakat et al. [20]	1	1	1	1	0	0	0	1	0	1	1	6
Barakat et al. [21]	0	1	1	1	0	0	0	0	0	1	1	5
Cordero et al. [22]	1	1	0	1	0	0	0	0	0	1	1	4
Cordero et al. [23]	1	1	1	1	0	0	1	1	0	1	1	7
Granath et al. [24]	1	1	0	1	0	0	0	0	0	1	1	4
Rodríguez-Blanque et al. [25]	1	1	0	1	0	0	0	1	0	1	1	5
Rodríguez-Blanque., et al. [26]	1	1	0	1	0	0	0	1	0	1	1	5
Rodríguez-Blanque et al. [27]	1	1	0	1	0	0	0	1	0	1	1	5
Rodríguez-Blanque et al. [28]	1	1	0	1	0	0	0	1	0	1	1	5
Sánchez García et al. [29]	1	1	1	1	0	0	0	1	0	1	1	6
Sillero et al. [30]	0	1	1	0	0	0	0	1	0	0	0	3
Smith and Michel [31]	1	0	0	0	0	0	0	1	0	1	1	3
Vallim et al. [32]	0	1	0	0	0	0	0	0	0	1	1	3
Vázquez-Lara et al. [33]	1	1	0	1	0	0	0	1	0	1	1	5

* This item is not used to calculate the PEDro score total.

## Data Availability

Data are available from the corresponding author upon reasonable request.
